# Biological and clinical review of IORT-induced wound fluid in breast cancer patients

**DOI:** 10.3389/fonc.2022.980513

**Published:** 2022-11-21

**Authors:** Shabnam Jeibouei, Forough Shams, Fariba Mohebichamkhorami, Davood Sanooghi, Bahareh Faal, Mohammad Esmaeil Akbari, Hakimeh Zali

**Affiliations:** ^1^ Cancer Research Center, Shahid Beheshti University of Medical Sciences, Tehran, Iran; ^2^ Future Foods Lab, Virginia Seafood Agricultural Research and Extension Center, Virginia Tech, Hampton, VA, United States; ^3^ Department of Medical Biotechnology, School of Advanced Technologies in Medicine, Shahid Beheshti University of Medical Sciences, Tehran, Iran; ^4^ Department of Tissue Engineering and Applied Cell Sciences, School of Advanced Technologies in Medicine, Shahid Beheshti University of Medical Sciences, Tehran, Iran; ^5^ Department of Biology, Central Tehran Branch, Islamic Azad University, Tehran, Iran

**Keywords:** breast cancer, IORT, seroma, personalized medicine, tumor microenvironment

## Abstract

Intraoperative radiotherapy (IORT) has become a growing therapy for early-stage breast cancer (BC). Some studies claim that wound fluid (seroma), a common consequence of surgical excision in the tumor cavity, can reflect the effects of IORT on cancer inhibition. However, further research by our team and other researchers, such as analysis of seroma composition, affected cell lines, and primary tissues in two-dimensional (2D) and three-dimensional (3D) culture systems, clarified that seroma could not address the questions about IORT effectiveness in the surgical site. In this review, we mention the factors involved in tumor recurrence, direct or indirect effects of IORT on BC, and all the studies associated with BC seroma to attain more information about the impact of IORT-induced seroma to make a better decision to remove or remain after surgery and IORT. Finally, we suggest that seroma studies cannot decipher the mechanisms underlying the effectiveness of IORT in BC patients. The question of whether IORT-seroma has a beneficial effect can only be answered in a trial with a clinical endpoint, which is not even ongoing.

## 1 Introduction

Breast cancer is the fifth most important reason for cancer death worldwide (1). Global statistics show that in 2020 female breast cancer caused 11.7% and 6.9% of new cases and deaths from all cancer types, respectively (2). Surgical intervention is the primary option for BC patient management. Based on prolonged research, the standard procedure is either an excision plus radiotherapy or a total mastectomy to achieve clear margins. It has been demonstrated that these two strategies are consistently equivalent in relapse-free and overall survival (3). In the early stages of BC, radiotherapy has been approved as a critical part of breast-conserving therapy (4). Following lumpectomy, radiation therapy is associated with fewer BC recurrences (distant or locoregional) and mortality (5). Hypofractionation and dose escalation were used as a standard of care. External beam radiotherapy (EBRT), which is typically administered in daily fractional doses during 5-6 weeks (45–50 Gy fractionated in 1.8–2.0 Gy per day), six weeks after surgery (6), while IORT is a high single dose of irradiation _either electrons (12Gy as boost dose/21Gy as radical dose) or X-rays (21Gy) _ given to the negative tumor margin during the surgery immediately after removing the tumor. Because electrons penetrate deeper than low-energy X-rays, breast tissue must be mobilized, and shields put into the posterior lumpectomy cavity to protect tissues inside the thorax. For measured depth, 20–21 Gy doses are regularly delivered at low-electron energy. Intraoperative electron radiation therapy (IOERT) is a common term for this method (7). Recently, the survival outcomes and local control of electron intraoperative radiotherapy (ELIOT) (using 50 kv IORT) and TARGIT (using 21 Gy IOERT) were released as two randomized clinical trials. They compared IORT and whole-breast EBRT (8, 9). TARGIT: means intraoperative radiotherapy with photon made by ZEISS COMPANY from Germany which is named “Intrabeam”. The dose of partial breast irradiation is 20 GY as Low KV-X Ray, which, based on biological and clinicopathological criteria is called: BOOST or RADICAL dose. ELLIOT: in completing different IORT procedures, here we are using Electron by two different doses: BOOST= 12 GY irradiation by an electron at the flap prepared during surgery that should be completed after surgery by EBRT. RADICAL: 21 GY irradiation by the electron during surgery as the radical dose which does not need EBRT anymore; it takes time less than 2 min. Moreover, the TARGIT-A trial showed risk-adapted targeted intraoperative radiotherapy (TARGIT-IORT) during lumpectomy for BC as impressive as whole-breast EBRT. TARGIT-IORT aims to achieve an accurately-positioned and accelerated form of tumor-bed irradiation, focusing on the target tissues alone, sparing normal tissues and organs such as lung, skin, heart, and chest wall structures from unnecessary and potentially harmful radiation treatment (10). Through ELIOT technique, the mobile linear accelerator delivers a single dose of radiation with electrons to the involved quadrant of the breast during surgery, reducing the radiotherapy course from six weeks to one single session during surgery (11). Both trials announced low local recurrence rates for IORT with tolerable toxicity and remarkable outcomes of overall survival (8, 9). In addition to these trials, emerging studies clarify the benefits and mechanisms underlying the local and systemic IORT in BC patients. Recently, wound fluid (seroma) has attracted the particular interest of researchers. It is typically formed in remained space after surgical excision. It leads to an inflammatory response in wound healing and seroma fluid accumulation in the subcutaneous area. During two recent decades, many studies have been done to clarify the impacts of seroma derived from IORT-treated tumor bed on the decrease of cancer recurrence. Studies by Belletti et al. and Herskind et al. have notified that seroma obtained from patients treated with IORT caused a reduction in proliferation and invasion of BC cell lines *in vitro* compared to seroma from non-treated patients.

Moreover, IORT treatment reported significant results in invasion (3-D Matrigel) and migration assays. No significant effects were observed on the proliferative capacity of seroma in 2D cell culture using BC cell lines (12, 13). Belletti et al. discovered an anti-cancer effect of TARGIT through changes in cytokines and growth factors in the resection cavity (12). Despite the promising results from these studies that introduced IORT-seroma as a tumor-inhibiting factor, there are many growing kinds of research on the analysis of seroma composition and effects of seroma on cell lines of BC and primary tissues that show contrary outcomes. Some of these studies performed using 3D *ex vivo *models have recently been performed by our research team. In this review, we will mention critical factors involved in BC recurrence, focusing on the direct and indirect effects of IORT on BC. Then we will discuss all the findings in this field of study to elucidate the benefit of removing or preserving IORT-seroma.

### 1.1 Factors involved in breast cancer recurrence

**Figure 1:**
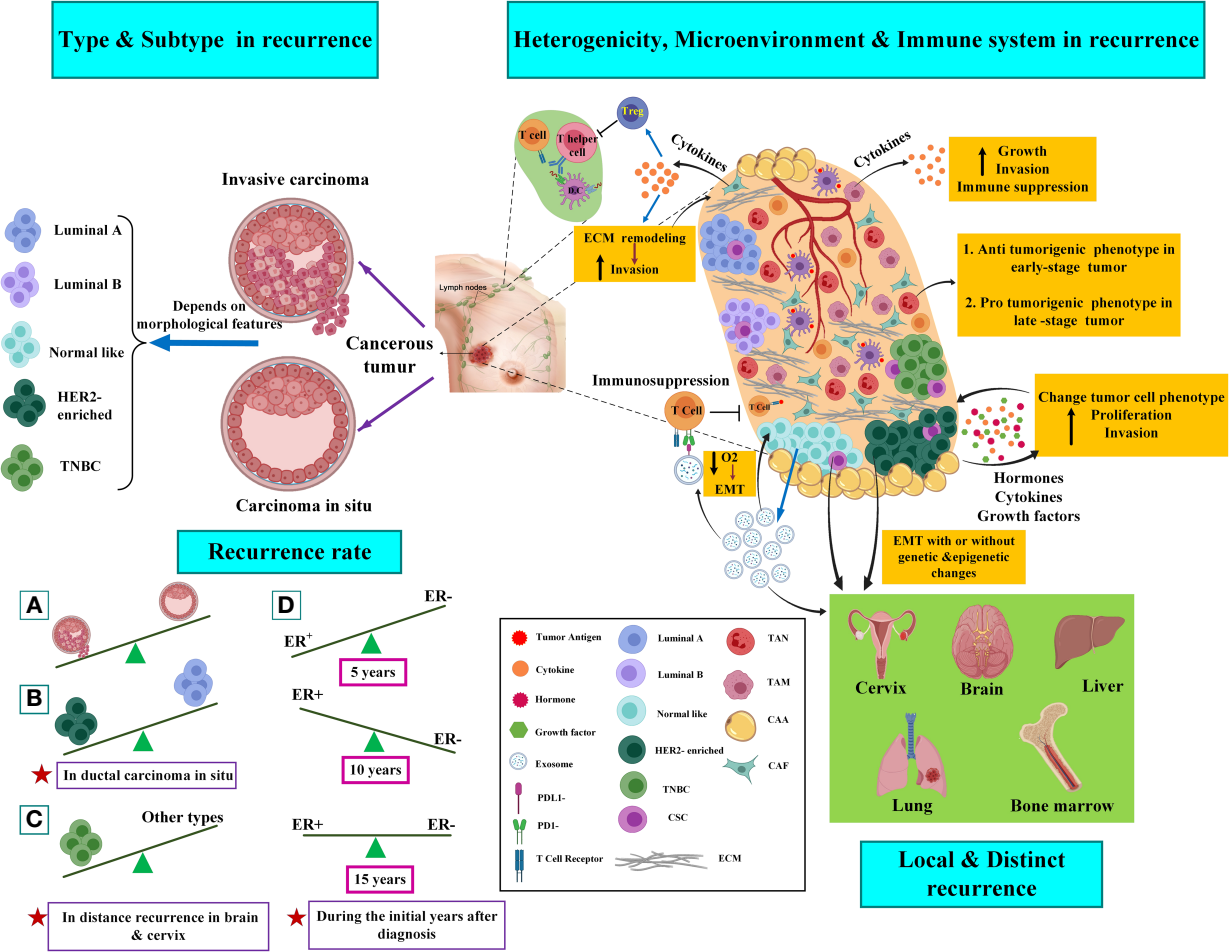
Factors involved in breast cancer recurrence. The left side of the picture shows the histopathologic types and molecular subtypes of the tumors in the recurrence of the disease. The right side of the picture shows the role of tumor heterogeneity, microenvironment, and immune system in breast cancer recurrence. Several clonal tumors containing CSCs are seen in tumor bulk and CSCs can affect the heterogenic migration of various clones in a single tumor. CSC also promote the EMT process and cause metastasis of BC tumor cell to the cervix, brain, liver, lung, and bone marrow. Cancer cells can recruit the immune system to progress tumor or even metastasis. Specific immune cells, including macrophages, lymphocytes, NK cells, dendritic cells (DCs), and neutrophils, are abundant and actively involved in the progression or suppression of cancer dissemination at the site of metastasis. Indeed, cancer cells can indirectly modulate and suppress the immune response (30, 32). CAFs promote tumor progression by initiating extracellular matrix remodeling through cytokine secretion. CAFs could suppress or avoid the immune response by promoting the recruitment of regulatory T cells (Treg), which is mediated by inflammation, or stopping the proliferation of T helper cells and killer T cells (28, 29). Work as tumor-modifying cells that may induce a change in cancer cell phenotype. CAAs produce hormones, growth factors, and cytokines. TAMs are the predominant immune cell types with immunosuppressive M2 polarized phenotypes that secrete tumor cytokines. Exosomes are essential in impairing both the adaptive and innate immune systems. It was shown that exosomal PDL1 derived from BC promotes and protects tumor growth by attaching to the PD-1 receptor of the CD8 T cells; thus, their adaptive killing activities are inhibited. Moreover, T-cells inhibit exosome secretion significantly through their anti-tumor immunity. In addition, uncontrolled cell proliferation induced by exosomes leads to inadequate nutrient and oxygen flow that derives the tumor microenvironment from becoming hypoxic. This process further triggers Epithelial-to-Mesenchymal Transition (EMT) and also promotes a more invasive phenotype. Further explanations are available in the text. TAN, tumor-associated neutrophil; TAM, tumor-associated macrophage; CAA, cancer-associated adipocyte; CAF, cancer-associated fibroblast; ECM, extracellular matrix; EMT, epithelial to mesenchymal transition; TNBC, triple-negative breast cancer; CSC, cancer stem cell. The figure was created using Biorender (https://biorender.com).

Ninety percent of all local relapses happen within proximity of the removed tumor site (14), and it may be due to remaining cancer cells in peritumoral tissue, which is developed by positive resection margins or perilymphatic and perivascular invasion (15). Studies showed that one of the important factors involved in BC recurrences could be the molecular subtype of the removed tumor. On the other hand, the heterogeneity of a single tumor results in drug resistance and recurrence. Moreover, the role of the microenvironment of the tumor bed and immune system in the development of recurrences would be significant. **Figure 1** schematically presents the factors that influence the recurrence of the disease.

#### 1.1.1 Molecular subtype

Different patterns of cancer recurrence have been suggested between various BC subtypes. According to **Figure 1** (left side), it seems that estrogen receptor (ER)-negative breast cancers are susceptible to higher recurrence during the first five years than ER-positive breast cancers following diagnosis. For the next ten years, the recurrence risk will chronically enhance in ER-positive breast cancers, and fifteen years after diagnosis, the risk seems to be equivalent for both subtypes. It has been demonstrated that in ductal carcinoma in situ, the human epidermal growth factor receptor 2 (HER2)-positive/progesterone** **(PR)-negative/ER-negative cancers showed a higher recurrence risk than HER2-negative/PR-positive/ER-positive cancers. Triple-negative breast cancer (TNBCs), which are typified by the absence of PR/ER/HER2, are commonly related to a higher risk of recurrence compared to receptor-positive tumors, particularly with a higher rate of recurrences in distant tissues (in the brain and visceral metastases) (16).

#### 1.1.2 Heterogeneity

Histopathologic, genetic, epigenetic, and single-cell sequencing studies, as well as the application of CTC-based assays in breast tumors, indicate that a single primary tumor can affect different regions and also, it may be able to phenotypic and genotypic change over time (17, 18). Tumor heterogeneity can be observed between cells within an individual patient’s tumor (intra-tumoral) or between cells of the same subgroup of tumors in different patients (inter-tumoral). At the genetic level, heterogeneity is connected to the copy number variation (CNV), down-regulation, and overexpression of a gene (due to missense, nonsense, or frameshift mutations) (19). Based on two concepts, including the cancer stem cell (CSC) hypothesis and the clonal evolution/selection model, primary single cells can undergo multiple molecular alterations and develop infinitive proliferative potential. The clonal evolution/selection model implies natural selection and explains how clones with higher epigenetic and genetic complexity can comply more under pressures than clones with low complexity (18). Unlike this model, the concept of CSC mentions self-renewal, capacity for clonal tumor initiation, and the potential for the clonal long-term repopulation (20). Interaction between CSCs and their niche (CSC surrounding microenvironment in a tumor) promotes invasion and metastasis of the tumor due to the production of factors, and the density of CSCs can affect the heterogenic migration of various clones in a single tumor (21, 22).

#### 1.1.3 Tumor microenvironment and immune system

Researchers are increasingly supporting evidence that acellular and cellular components in the tumor microenvironment (TME) play a principal role in tumor growth and response to treatment. Cancer-associated fibroblasts (CAFs) are the main components in cancer stroma and TME. They promote tumor progression by initiating extracellular matrix remodeling through the secretion of cytokines (23). Adipocytes are other cell types that form TME. The most abundant component covering the cells in breast tumors is adipose tissue. Cancer-associated adipocytes (CAAs) work as tumor-modifying cells that may induce a change in cancer cell phenotype (24). Adipocytes produce hormones, growth factors, and cytokines. However, their role in the expansion of BC has not been fully discovered yet (25). Numerous types of immune cells, including macrophages, various phenotypes of T cells, B lymphocytes, natural killer (NK) cells, mast cells, and neutrophils, are found in breast tumors as part of normal tumor anatomy (26). Tumor-associated macrophages (TAMs) are the predominant immune cell types with immunosuppressive M2 polarized phenotypes that secrete tumor cytokines (IL-4, IL-10, and IL-13). These cytokines promote tumor growth by stimulating immune cell differentiation into mature macrophages (27). The interaction of tumor cells and the matured macrophages cause the secretion of various factors, such as vascular endothelial growth factor (VEGF) and colony-stimulating factor–1 (CSF1), *via* tumor cells, promoting tumor growth and invasion (28).

TAMs, through expressing heme oxygenase-1 (HO-1), an enzyme that inhibits immune system, suppress the endothelial cells’ response to tumor necrosis factor-a (TNFα), an immunogenic cytokine, and then maintain the immunosuppressive tumor microenvironment (29). Cancer progression and anti-cancer response also depend on interactions between cancer cells and the immune system. The interaction can be categorized into four groups: First, the process of immunosurveillance; second, the anti-cancer immune response; third, immunosuppression; and fourth, the cancer assistance program. Through the immunosurveillance mechanism, the healthy immune system continually checks tissues for the manifestation of cancer, particularly the existence of tumor antigens, including abnormally expressed, mutated, or oncoviral proteins. Following prosperous detection of tumor cells, the anti-cancer immune responses proceed and are identified by killer cells and T helper cells and then lyse the cancer cells. Furthermore, cancer cells can indirectly modulate and suppress the immune response. The c activation of b-catenin mediated by cancer cells in DCs is a specific example of this process.

DCs are responsible for presenting killer T cells adhered to the particular tumor antigens with the ability to direct the anti-tumor immune response. DCs cause a repressed cross-priming of CD8+ T cells adhered to tumor antigens following high levels of activated b-catenin; therefore, the entire process of anti-tumor immune response mediated by CD8+ T cell is dampened (30). Then, the immune cells can have a dual function in the tumor microenvironment; while specific features of tumor stroma can trigger immune cells to develop tumor suppression, other signals and features of the tumor can promote immune system-mediated tumor invasion (31–34).

Among acellular components of TME, exosomes possess a crucial role in shaping the microenvironment of the local tumor through paracrine crosstalk between stromal cells and tumor and in organizing future sites of metastasis. Exosomes are small extracellular vesicles with an average size of 100 nm with an endosomal origin that deliver various types of molecular and genetic information (e.g., lipids, proteins, and nucleic acids) to neighbor and distant cells. In pioneering studies, David C. Lyden et al. demonstrated that exosomes have a critical role in pre-metastatic niche formation in distant organs. Furthermore, the studies highlighted the role of exosomal integrins in directing organotropic metastasis. These findings bring further insight into cancer development’s complexity while demonstrating the existing gaps in our knowledge (35).

### 1.2 Responses of tumor and tumor bed to IORT

**Figure 2:**
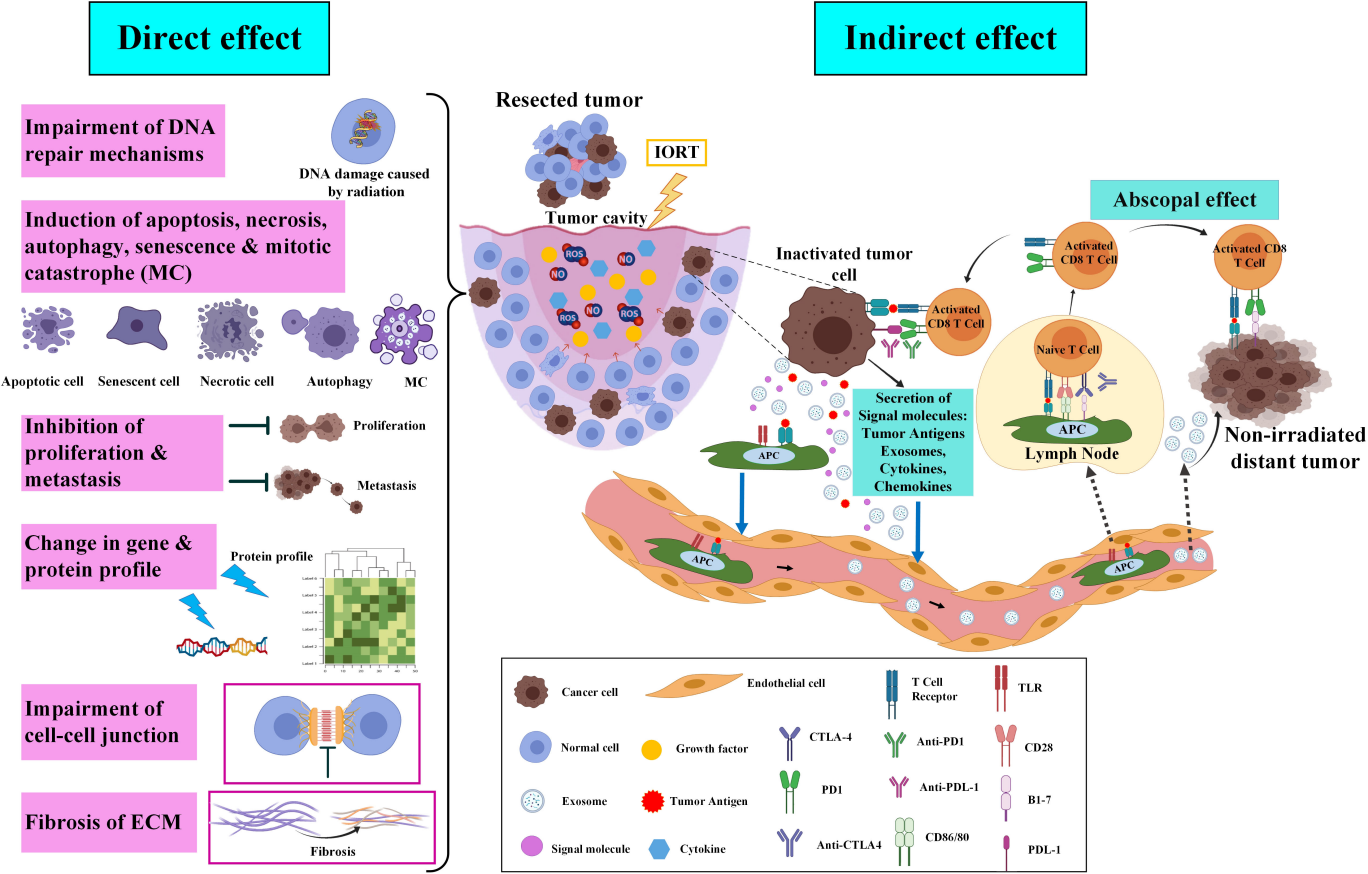
Direct and indirect effects of IORT on cancer inhibition and recurrence. After the breast-conserving surgery, molecular and probably remained cancer cells in the tumor cavity will be affected by IORT. Briefly, irradiation on the tumor cavity impairs DNA repair mechanisms, induces cell death, inhibits proliferation and metastasis, alters gene and protein profiles, impairs the cell-cell junction, and induces ECM fibrosis. In indirect effects of IORT, inactivated remained tumor cells release exosomes and tumor antigens which move and deliver to lymphatic vessels through the circulating system and APCs, respectively. IORT-induced tumor antigens and exosomes effects non-irradiated tumor cells in the body via a mechanism named “abscopal effect” (33). The figure was created using Biorender (https://biorender.com).

IORT is used in a high single dose that targets the wound cavity with a higher recurrence risk while spars the surrounding tissue and providing acceptable cosmetic and toxicological results (36–39). The IORT with direct effects removes survived tumor cells in the margin and non-irradiated neighbor cells through the bystander effect. The tumor microenvironment also receives significant modifications (39).* *Discovered direct and indirect biological responses of probably remained tumor cells as well as tumor bed cells to IORT are represented here and schematically presented in **Figure 2**.

#### 1.2.1 DNA repair mechanisms

Mainly, due to less time to repair their damages, IR induces more efficient cell death in proliferative cells than quiescent cells. Oxygen concentration is decreased in tumor tissue, and it was demonstrated that poor-oxygenated cells are less sensitive to IR radiation compared to those well-oxygenated cells. Cancer cells are more susceptible to unrepaired damage due to faster proliferation than normal cells; they often have several mutations that cause continual stimulation of the repair process. It can lead to their survival from damage; however, it may lead to the death of surrounding normal cells (40–42).

#### 1.2.2 Metastasis, proliferation, and metabolism

After BC surgery, IORT targets tumor bed cells through modifying their growth conditions, such as preventing mammary mesenchymal stromal cells (MSC) from outgrowth after IORT (43). Segatto et al. supported this concept by finding the expression of miR-223 in the cells of the tumor bed that undergo IORT. This expression causes the reduction of epidermal growth factor (EGF) expression that finally abrogates cell growth and tumor recurrence (44). The main signaling pathway mediated by growth factors responsible for cancer cell survival and proliferation is the PI3K/AKT/mTOR (45), in which we recently observed downregulation of proteins that are part of the PI3K-Akt signaling pathway led to disrupted proliferation following radiation (46, 47). The tumor margin of cancer after 24h following IORT showed downregulation of central carbon metabolism. Cancer cells alter their metabolism to promote unrestricted cellular proliferation and respond to energetic and biosynthetic demands (48). Warburg first discovered that cancer cells have more demands for glucose; then, the glycolysis process is increased in them (49). After irradiation, processes associated with Glycolysis/Gluconeogenesis were suppressed, and several carbon metabolisms, including citrate cycle and fatty acid and amino acid degradation, were activated. After IORT, changes in the metabolisms could aid the environment in infiltrating immune cells and restrict the proliferation of remaining tumor cells (50).

#### 1.2.3 Cell death mechanisms

Growing evidence shows that triggering the cell death mechanism as a therapeutic effect of radiotherapy would be a complicated process. Notably, nowadays, it is detected that suppression of the proliferative capacity of tumor cells behind irradiation happens through various mechanisms, including apoptosis, necrosis, senescence, autophagy, and mitotic catastrophe (MC) (51, 52).* *In high LET (linear energy transfer) radiation, direct ionization of cell macromolecules such as DNA, RNA, proteins, and lipids induce cell damage. Radiation with low LET leads to indirect damage to these macromolecules because of reactive oxygen species (ROS) production, particularly hydroxide and superoxide radicals produced from the radiolysis process of reactive nitric oxide species (RNOS) and intracellular H2O. These sources of ROSs trigger several intracellular signaling pathways and oxidate macromolecules, which lead to inflammation and stress responses (53–57). IR stimulates pro- and anti-proliferative signaling pathways that unbalance cell decisions about survival/apoptosis (58), which are regulated by several factors and genes involved in DNA repair, inflammation, cell cycle progression, and cell survival/or death (58–61). Altogether, various kinds of tumor cells, including immortalized keratinocytes, lung, colon, and prostate cancer, experience apoptosis after IR radiation (from 1 to 20 Gy). Many non-immortalized cells require higher irradiation doses (>20 Gy) to show apoptosis (51, 62, 63). Moreover, several data support the notion that IR treatment may stimulate p53-independent apoptosis by the mechanism of the membrane stress pathway, *in vitro* and *in vivo*, by sphingomyelin transmembrane signaling through the production of ceramide second messenger (57, 64). Radiation-triggered necrosis, unlike apoptosis, is mainly linked with intensified inflammation in the surrounding normal tissue (65). Necrosis is a type of cell death that has commonly been highlighted as a consequence of high IR doses (32 to 50 Gy) (57, 66). Senescence is a recognized procedure throughout aging and tissues under IR-treated or several stress stimuli such as chemotherapeutic agents, oxidative stress, and prolongation of signaling through some cytokines. The senescent cells can consequently initiate the pathology process (67–70). The primary cell response of lower doses of IR is senescence, while higher doses of IR induce necrosis or apoptosis in the same cells. In a study, X-ray irradiation of endothelial cells of the pulmonary artery showed that an intensifying dose of IR radiation induces a range of cell responses from senescence in lower doses and autophagy/apoptosis and necrosis at higher doses (62). Much data have been proposed that senescence in cancer cells-treated with IR could cause a decrease in self-renewal capacity (71, 72). Despite the potential tumor suppression role of senescent cancer cells, they could secrete a particular profile of the Senescence-Associated Secretory Phenotype (SASP), which contains a various range of cytokines, proteases, and growth factors. SASP changes the tissue microenvironment and triggers tumorigenesis and angiogenesis, as well as the EMT process and invasion. Although, SASP has anti-tumor function through tumor cell clearance *via* the immune system (73–75). Autophagy is a primary catabolic mechanism of cell degradation through lysosomal action that lyses dysfunctional or unnecessary cell components (76). Autophagy is a procedure to maintain metabolic homeostasis in tumor cells undergoing nutrient depletion and chronic hypoxia (41). This pathway can stimulate survival or cell death in IR-treated cells; the mechanisms depend on the gene expression controlling apoptosis, which is also cell and tissue-specific (77, 78). Mitotic catastrophe (MC) is associated with different biochemical and morphological changes. A cell death process occurs after or during aberrant mitosis and incomplete DNA synthesis following radiation (79). Checkpoint deficiencies in tumor cells cause defective DNA replication and malfunctioned repair mechanisms in the aberrant segregation of chromosomes that may lead to MC. Therefore, the control loss of checkpoints in IR-treated cancer cells may generate aneuploid progeny and cell death due to MC (57).

#### 1.2.4 Cell-cell and cell-(extracellular matrix)ECM interaction

Crosstalk between PI3K/AKT/mTOR signaling pathway mediated by growth factors and cell adhesion to the ECM activates several vital biological processes, such as regulation of gene expression as well as proliferation, differentiation, survival, and motility of cells (80, 81). We also found a number of downregulated proteins through analysis of the KEGG pathways 24h after indirect irradiation of the tumor margins of breast-conserving surgery (BCS) plus IORT. The proteins are involved in the processes associated with focal adhesion, ECM-receptor interaction, and Rap1 signaling pathway (46, 82).

#### 1.2.5 Gene and protein expression profile

Despite many significant research interests in the scientific community about the clinical application of IORT on different types of cancers, a limited number of papers were concerned about induced gene expression following treatment with IORT. A recent study on BC cell lines (both tumorigenic and non-tumorigenic cell lines) exposed with doses of 10 and 23 Gy identified differences among various types of cell lines and treatment after using microarray for gene expression profiling (83). According to our previous omics investigations on the tissue of negative tumor margins, radical and boost doses of IOERT change different molecular pathways (82). They could stimulate the activity of some signaling pathways, such as nuclear factor kappa B (NF-κB), TNF, forkhead Box O1 (FoxO), and hypoxia-inducible factors1 (HIF-1). We also detected that apoptosis, B cell receptor, Toll-like receptor, and metabolic pathways were upregulated, known to have systemic and local effects. The proteome profile was obtained from the isobaric tag for relative and absolute quantitation (ITRAQ) technique of tumor margin samples of patients under treatment with IOERT, 21Gy (sample collected before and 24 h of after treatment with IOERT). According to our results, the tumor margin samples collected before and after IORT showed alterations in the expression of many genes and enhanced pathways linked to cell growth, survival, programmed cell death, and cell cycle arrest. In addition, downregulated proteins that were part of the phosphatidylinositol 3-kinase (PI3K)-AKT signaling pathway showed disruption of proliferation after IORT (82). Besides, inhibition of this pathway, directly and indirectly, could influence the radiation results (82).

#### 1.2.6 Abscopal effect

Local IR treatment of tumors often results in systemic responses at distant sites. This phenomenon is termed the “abscopal effect,” which induces and enhances the innate and adaptive immune responses against tumors (84). The abscopal effect is an antitumor consequence of radiation that can be seen in metastatic conditions away from irradiated tissue (85). In radiation therapy, diverse mechanisms are associated with the abscopal effect. Generally, the mechanisms include increasing the lymphocyte infiltration into the tumor’s microenvironment, improving detection and tumor cell death by enhancing the tumor’s antigens expression and antigen presentation machinery, increasing tumor sensitivity, and activating ascending modulatory pathways. The radiation-induced abscopal effect depends on the immune system through various strategies. In some cases, radiation therapy can activate the host immune system, especially in immunogenic cancers such as melanoma, hepatocellular carcinoma, and renal cell carcinoma. The synergistic effect between the host immune response and the abscopal effect induced by radiation therapy stimulates antitumor effects against micro-metastases beyond the irradiated area. A combination of immunotherapy and radiation therapy is used in some other cases, mainly involving less immunological cancers such as BC. Thus, immunogenic reagents, including immune checkpoint blockers and targeted immunomodulators, are combined with radiation therapy in this type of cancer. This combination promotes the host immune response against tumor cells and stimulates the abscopal effect after radiation therapy (86). However, in a rare case, Azami and colleagues reported that local radiation monotherapy in advanced BC, with extensive lymph node, lung, and bone metastases, effectively induced an abscopal effect in non-irradiated metastatic regions. A few months after radiation therapy, they observed that metastatic lesions regressed in the irradiated breast tumors and all non-irradiated areas (87). Generally, in the first antitumor strategy, high-dose radiation therapy with a direct effect on tumor cells kills these cells. It releases the remnants of dead tumor cells that contain potentially immunogenic molecules. These factors stimulate the immune system and lead to immunological cell death through T-regulatory cells, DCs, and suppressive cells (88). The combination of radiation therapy and granulocyte-macrophage colony-stimulating factor (GM-CSF) in some solid metastatic cancers can induce an abscopal effect and increase the overall survival of patients. Formenti and colleagues showed that in metastatic BC, the combination of radiation therapy with a systemic transforming growth factor-β (TGF-β) blocking antibody called Fresolimumab induces a dose-dependent systemic immune response and improves overall survival (89).

#### 1.2.7 Effect of drainage on clinical outcome

Several studies have explored the safety of seroma drainage according to multiple clinical endpoints (**Table 1**). Quality of life has been reported in various kinds of results in different studies. Better quality of life was seen in long-term drainage (90), reduction of hospital stays, and early drain removal in some studies provided a better quality of life by decreasing post-operative complications or in some studies reported no adverse effects, so early drain removal was preferred by patients (98, 99). To the best of our knowledge, in IORT, no results were reported regarding drainage tube removal time, length of hospitalization, and post-operative complications. However, IORT had promising results, both in terms of saving healthy tissue and local control (74% to 100% at 5 years) and 96.2% disease-free survival (101, 103). The local relapse prompted a series of clinical trials and studies to investigate whether localized IORT could be as efficient at preventing recurrence at local site as standard postoperative radiotherapy of the whole breast while also being more patient-friendly in terms of decreasing the treatment duration. As we mentioned earlier, IORT decreases the possible risk of tumor cell repopulation during the wound healing process through direct radiation therapy of diseased tissue within the tumor bed during the surgical procedure (9).

**Table 1:** Evidence Summary of drain policy and its clinical effects.

	Patient Numbers	Standards are regarding drains (days after surgery)	Standards are regarding drains (volume of seroma)	Influence on infection rate or wound healing	Improves quality of life or not	Outcome	Ref
**surgery**	88	24 hours compared to 5 days after surgery	Higher seroma formation in the 1-day group (161.25 ml) compared to the 5-day group (7.50 ml) one week after surgery	The lower frequency of wound infection in the long-term compared to short-term drainage	Quality of life is better in the long-term (5 days) drainage	long-term drainage reduces the risk of seroma formation compared to short-term	Jafari Nedooshan J et al., 2022 (90)
	187	24h after surgery	Three groups (10ml, 20ml, 30ml)	Wound infection was similar at different drain removal times	Yes, the Significantly better quality of life in the 20 mL group	The 20 mL group had relatively low postoperative complication rates	Wen N et al., 2022 (91)
	40	off-drain day I compared to day III post-surgery	Mean 157.31 ml in 1-day compared to 149.58 ml in 3-day post-operative drain removal	–	Early drain removal reduces the symptoms felt by breast cancer patients related to drain.	Early drain removal does not reduce seromas incidence seven days after discharge	Ramadanus et al., 2022 (92)
	A meta-analysis including 11 RCTs	Early drain removal was defined between postoperative days 1-7 and late drain removal was dependent on the output	20mL/24h to 50mL/24h (The mean total seroma formation was 326 mL in 3 d)	Early removal did not increase surgical site infection	Early drain removal has no proven clinical benefit except the reduction of hospital stays	Early drain removal shortens hospital stay length while increasing the risk of seroma formation	Shima H et al., 2021 (47)
	88	The Redon drain and the Quadrain drain with a mean duration of drains *in situ* 42.6* h* and 50.1 h respectively	The standard of drain removal was less than 30ml/24h. mean volume was 12.3 ml for the Redon drain and 13.0 ml for the Quadrain drain (Not different for both drains)	No difference in surgical site infections between the two groups	Did not differ concerning either efficacy or safety	Not significantly different concerning duration in the surgical site, post-operative pain, seroma volume, and cosmetic result	Schmidt G et al., 2019 (93)
	202	The mean postoperative day of drain removal is 14 days (9 patients had no drainage in a surgical modality)	Drain removed when the drainage fluid volume was 20 ml or less per day and the total volume was 1456 ml	Relative higher risk of seroma infection without drainage	surgical modality affected the quality of life post-operation	A high rate of seroma formation and prolonged fluid discharge were observed without drainage	Isozaki H et al., 2019 (94)
	251	Two groups, including quilting sutures with and without wound drainage	–	The incidence of postoperative infection significantly decreased without postoperative drain	- (Postoperative drain could be omitted based on the operation technique)	The group without a postoperative drain had lower seroma incidence and wound complications compared to the group with a drain	Ten Wolde B et al., 2019 (95)
	99	Early removal (4–5 days postoperative) compared to output-based drain removal when	Less than 30 ml/day in the early removal group. Total volumes of fluid drained were significantly lower in the early-removal group (median 752 ml versus 1745 ml)	No negative influence on the wound infection rate in the early removal group	Yes, Early drain removal was associated with a significant improvement in quality of life	Early drain removal has no negative effect on clinical outcomes with considerably lower home care nursing	Vos H et al., 2018 (96)
	214	Average day 5 (1–5) postoperative as the study group and Day 6 (3–15) as the control group	Drain removal when was less than 50 ml/24 h. Mean total volume was 351 ml in the study and 416 ml in the control group	No significant difference between the two groups	Yes, no adverse effect on the quality of life in early drain removal	Early drain removal is safe with a shorter hospital stay despite the slightly increased chance of seroma formation	Okada N et al., 2013 (97)
	87	Early (day 4) to late (day 10) drain removal	Drain removal when was less than 30 ml/24h. Total drainage volume was significantly higher (1123ml) in late than those with early drain removal (571 ml)	The lower wound infection rate in early removal	No negative effect on the quality of life in early drain removal so it was preferred by patients	Shorter hospital stay and slightly higher risk of seroma formation in early drain removal	Clegg-Lamptey JN et al., 2007 (98)
	100	Short (24 h) versus long-term (up to 7 days) postoperative drainage	Drainage removal when was less than 50 ml/24h. No significant difference was seen in the mean volumes of aspirations (213 ml in short vs 186 ml in long-term drainage)	Lower Infectious complications in short-term drainage	Yes, short-term drainage provided a better quality of life by decreasing post-operative complications	Short-term (24 h) drainage was associated with a shorter hospital stay, a higher risk of seroma formation, and lower wound-related complications	Baas-Vrancken Peeters MJ et al., 2005 (99)
	121	5-day vs. 8-day groups	Drain removal when was less than 30 ml/24h. no significant difference in the volume of seroma drainage between the two groups	No negative effect on the wound infection in both group	Yes, 5-day postoperative drain removal allowed for better utilization of community resources without adversely impacting patients’ physical or psychological welfare or outpatient facilities	Five-day post-operative drainage was as safe as 8-day however increased the risk of seroma formation requiring aspiration	Gupta R et al., 2001 (100)
**IORT**	797	- IOERT vs whole breast irradiation groups	179 patients (22.46% of cases) who developed seroma	Surgical wound infection in one patient	Yes, by providing long-term efficacy and acceptable cosmetic result	IOERT-boost improves local control with 96.2% disease-free survival and reduces local recurrence at long-term follow-up (Mean 5 years)	Ciabattoni et al., 2022 (101)
	160	5.9 days in IORT^+^ versus 5.0 days in the IORT^-^ groups	No difference between groups in incidences of seroma	No difference in infection	Yes, IORT safely delivers radiation therapy with acceptable acute toxicity, is well-tolerated	No difference in terms of drainage tube removal time, length of hospitalization, and postoperative complications	Hu X et al., 2020 (102)
	90	- IORT vs TARGIT E groups	Seroma formation occurred in 15 patients (16.5% of cases)	15 patients (16.5% of cases) had an infection	Yes. IORT had promising results in saving healthy tissue and local control	In the IORT group, overall survival was 100% After a median follow-up of 27.4 months, and the local recurrence rate was 2.4%	d’Illiers et al., 2018 (103)

### 1.3 Studies associated with effects of IORT-seroma on breast cancer progression

**Figure 3:**
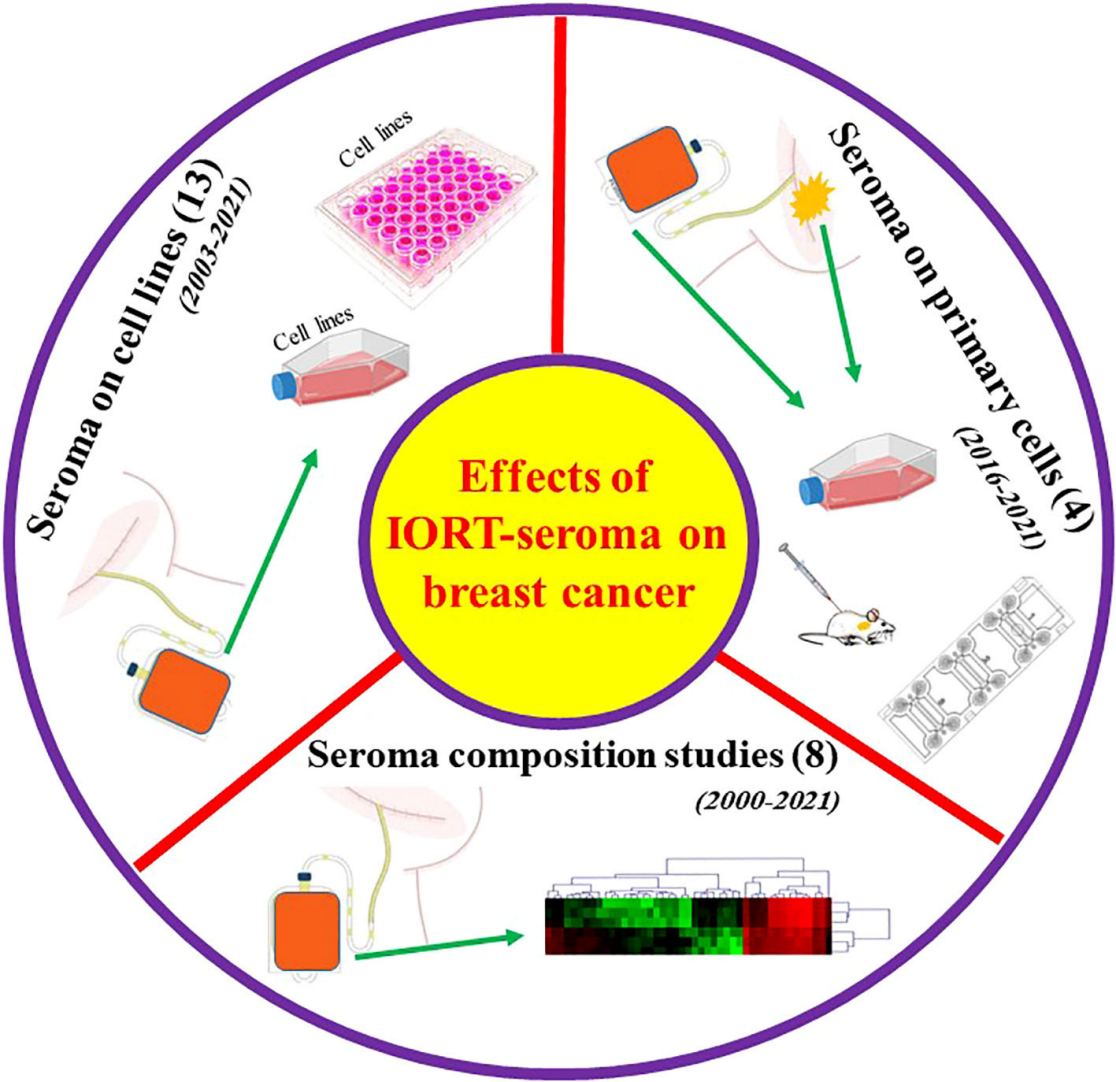
Graphical abstract for performed studies in breast surgery IORT- and non-IORT-seroma and their effects on breast cancer.

Key information related to all the studies about breast surgery IORT- and non-IORT-seroma and their effects on BC (according to our knowledge) is available in **Table 2**. See also **Figure 3**.

**Table 2:** Summary of studies associated with effects of seroma and IORT-seroma on breast cancer.

	Method	Results	Author	Year
**Seroma composition**	Hematological and biochemical analysis of 3 or 4-day seroma from 18 BC patients undergoing mastectomy with complete axillary clearance or wide local excision.	Reflection of the exudative phase of wound healing in seroma.	McCaul et al.	2000
	Quantitative assessment of CEA and CK-19 in 24h seroma from 126 BC patients.	The high sensitivity of CEA and CK-19 for detection of locoregional recurrence in BC patients.	Zhang et al.	2006
	Wound fluid injection near the tumor site in syngeneic BC xenografts in mice.	Enhanced tumor growth.	Christina et al.	2008
	Evaluating proteomic profile of 24h seroma from 45 BC patients.	Increase of 10 and decrease of 20 tumor progression associated proteins in IORT-seroma compared with non-IORT-seroma.	Belletti et al.	2008
	Assessment of 80 cytokines, chemokines, and growth factors in 1 or 2-week seroma from 59 patients with benign or malignant lesions.	Increased expression levels of key tumor-triggering cytokines and decreased expression of important tumor-inhibiting factors in seroma from BC patients compared to seroma collected from non-cancer patients.	Valeta-Magara et al.	2015
	Assessment of 34 chemokines, cytokines, and growth factors in 24h seroma collected from 27 BC patients.	Association of the composition of seroma with molecular features of the excised tumor.	Agresti et al.	2019
	Quantitative analysis of the factor composition of 48h seroma from 38 BC patients.	Decreased level of IL-7, IL-8, MIF, IL-13, and TNF-beta and increased level of CTACK, HGF, G-CSF, TNF-alpha, and IL-1 beta in IORT-seroma compared with non-IORT-seroma.	Kulcenty et al.	2019
	Analysis of immune cell populations and cytokines in 24h seroma from 42 patients.	No significant difference in cell count between IORT group and control. Increased level of Leptin and decreased level of GRO-α, IL-1β, and Oncostatin-M in IORT group.	Wuhrer et al.	2021
**Seroma on cell lines**	Evaluating proliferative effects of 24h seroma from 13 BC patients on SKBR-3, MDA-MB361, MDA-MB-453, MDA-MB-231, MDA-MB435, and MCF-7 cell lines in 2D system.	Induction of proliferative effects in all the cell lines.	Tagliabue et al.	2003
	Evaluation of cell growth and motility in MCF-7, T47D, MDA-MB-453, MDA-MB-231, and SKBR-3 cell lines under 24h seroma from 45 BC (IORT and non-IORT) patients in 2D and 3D systems.	Stimulation of proliferation, invasion, and migration of BC cell lines under seroma treatment. Abrogated stimulatory effects under IORT-seroma treatment.	Belletti et al.	2008
	Evaluation of proliferation in MCF-7, HCC1937, and under treatment of 24h or 48h seroma from 30 patients (in 3 groups) in 2D system.	Induction of proliferation in HCC1937 and MCF-7 in a similar manner.	Ramolu et al.	2014
	Evaluation of clonigenic and long-term proliferation effects of 24h seroma from 30 BC (IORT and non-IORT) patients on MCF-7 cell line in 2D system.	No significant difference between IORT- and non-IORT-seroma groups.	Veldwijk et al.	2015
	Evaluation of cancer stem cell phenotype in MDA-MB-231, BT-20, MDA-MB-468, SK-BR-3, BT-549, BT-474, MCF7, and T47D cell lines under seroma treatment from 44 BC patients (IORT and non-IORT).	Decreased CSC population in IORT-seroma affected in cell lines of MDA-MB-468 and BT-549. Inhibition of CSC populations in both IORT- or non-IORT-seroma affected MCF-7 cell line.	Zaleska et al.	2016
	Evaluation of mammosphere formation in BT-474, MDA-MB-231, MDA-MB-468, and MCF-7 cell lines under treatment of 24h seroma from BC patients in 2D system.	Stimulation of mammosphere formation and also STAT3 activation.	Segatto et al.	2018
	Evaluation of apoptosis pathways in MCF-7 cell line under treatment of 7-day seroma from BC patients (IORT and non-IORT).	Activation of extrinsic apoptosis pathway by IORT-seroma.	Kulcenty et al.	2018
	Evaluation of proliferation and migration of MDA-MB-231, HCC1937, BT-549, SKBR-3, T-47D and, MCF-7 under 24h seroma treatment from 27 BC patients in 2D system.	Stimulation of proliferation and migration in all the cell lines over 4 days.	Agresti et al.	2019
	Measurement of the level of breaks double-strand DNA, apoptosis induction and the changes in DNA repair associated gene expression in MDA-MB-468 and MCF-7 cell lines under 48h seroma from 16 BC patients (IORT and non-IORT) in 2D system.	Induction of breaks in double-strand DNA and enhanced expression of DNA repair-associated genes in IORT-seroma group.	Piotrowski et al.	2019
	Evaluation of changes in CSC phenotype and EMT in MCF-7 and MDA-MB-468 cell lines under 48h seroma from 16 BC patients (IORT and non-IORT) in 2D system.	Stimulation of phenotype of CSC and EMT process in non-IORT group and abrogation of them in IORT group.	Kulcenty et al.	2019
	Microarray analysis of biological processes in MDA-MB-468 under 48h seroma from 43 BC patients (IORT and non-IORT) in 2D system.	Common biological processes in both IORT- and non-IORT groups.	Kulcenty et al.	2020
	Evaluation of behavior and secretome of MDA-MB-231 and mesenchymal stromal cells under 24h seroma from 42 BC patients (IORT and non-IORT) in 2D system.	Reduced proliferation of MSCs, capacity of wound healing and activity of chemotactic migration under IORT-seroma treatment.	Wuhrer	2021
	Evaluation of viability, proliferation, migration and invasion in MCF-7, MDA-MB-231, and SK-BR-3 cell lines under treatment of 24h seroma from 20 BC patients (IORT and non-IORT) in 2D system.	Decreased number of colonies in IORT-seroma affected MCF-7 cells. No significant difference between two groups in expression levels of P21, P16 and Cas3.	Jeibouei et al.	2022
**Seroma on primary cells**	Evaluation of survival rates in cells from human-derived BC cells under 3-day 21 seroma treatment in 2D system.	Increased survival rates and promote drug resistance in seroma-treated cells.	Zhang et al.	2016
	Evaluation of proliferation and migration in human-derived BC tumor spheroids from 4 specimens under seroma treatment from the patients in 3D microfluidic system (IORT and non-IORT) using time laps imaging.	Increased proliferation and migration rate in IORT-treated group compared with control.	Javadi et al.	2021
	Evaluation of cell viability of human-derived BC tumor spheroids from 23 specimens under seroma treatment from the patients in 3D microfluidic system.	Induction of cell viability in 22 specimens under seroma treatment compared with control. Inhibition of cell viability under seroma treatment in 1 specimen compared with control.	Jeibouei et al.	2021
	Evaluation of cell viability and measurement of the expression levels of apoptosis and migration/invasion-related proteins in human-derived BC tumor spheroids from 20 specimens under treatment of seroma from the patients (IORT and non-IORT) in 3D microfluidic system.	No significant difference in the percentage of live cells in IORT-seroma and non-IORT-seroma groups. No significant difference in Cas3 expression level between two groups. Higher level of E-cad expression in IORT group.	Jeibouei et al.	2021

Studies are divided into 3 sections, including seroma composition studies, effects of seroma on BC cell lines, and studies on the effects of seroma on primary tumor tissues.

#### 1.3.1 Composition of breast surgery-induced seroma; benign, malignant, IORT-treated

**Figure 4:**
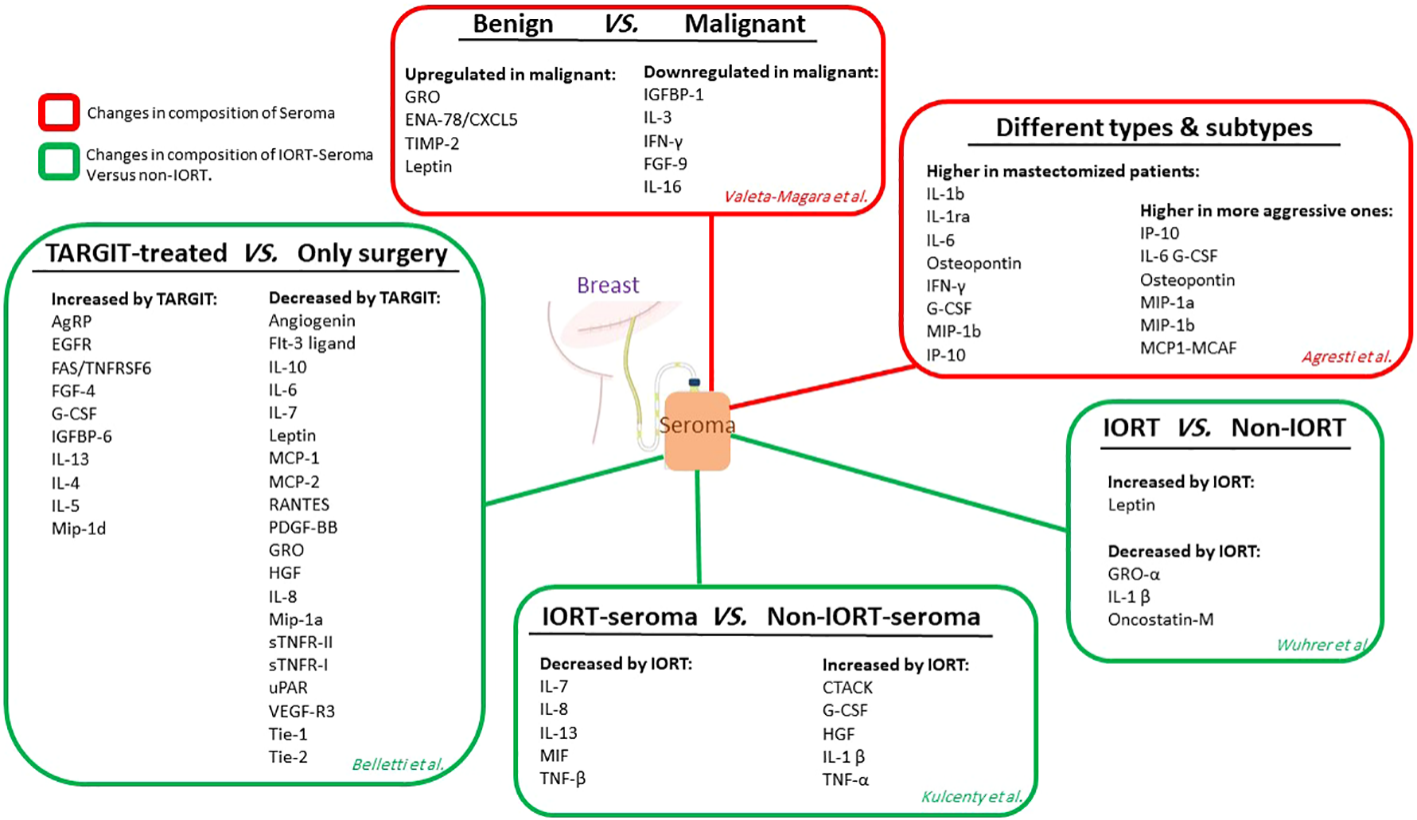
The studies related to seroma composition. Red boxes show the level of protein expression in seroma without considering radiotherapy. Green boxes show protein expression levels in an IORT-affected seroma compared to non-IORT-affected seroma.

Seroma is an inflammatory exudate most commonly found during the first step of wound healing (104). It has been illuminated that seroma inoculation near the tumor site in mice with syngeneic BC xenografts led to enhanced tumor growth (105). Seroma derived from surgical sites may show brief information about the cell activity in terms of the release of growth factors, chemokines, and cytokines that are vital in repair and healing (106, 107). The differential expression of pro-oncogenic growth factors and cytokines is secreted from malignant to benign lesions in the post-surgical seroma in breast tumors. Valeta-Magara and colleagues detected that seroma from the surgical cavity of BC patients expresses a higher level of fundamental tumor-promoting cytokines. In contrast, benign surgical lesions in non-cancer patients express a lower level of principal tumor-inhibiting factors. They assessed 80 different cytokines, growth factors, and chemokines in 59 post-surgical seroma (24 patients with benign and 35 with malignant lesions). Although the results showed that 28 cytokines were overexpressed in both groups of seroma. Malignant-derived seroma showed higher expression of 9 biologically important factors. In particular, Leptin, tissue inhibitor of metalloproteinases 2 (TIMP-2), growth-regulated protein (GRO), and epithelial neutrophil-activating peptide 78/chemokine (C-X-C motif) ligand 5 (ENA-78/CXCL5) were highly overexpressed in malignant seroma. At the same time,insulin-like factor binding protein-1(IGFBP-1), IL-3, IL-16, fibroblast growth factors-9 (FGF-9), and IFN-γ showed down-regulation in malignant compared to the benign seroma. The post-surgical cavity of a breast tumor contains pro-inflammatory factors, regardless of being malignant or benign; however, in malignant tumors, a higher amount of additional pro-oncogenic cytokines, chemokines, and growth factors and a reduction in tumor-inhibiting factors are detected. These results showed the preconditioning effect of normal surrounding tissue on the tumor and provided a pro-oncogenic environment that remains after the removal of the tumor by surgery (108). In a recent study, Agresti and collaborators detected 34 cytokines, growth factors, and chemokines in seroma of 27 BC patients that promote the initiation and development of cancer. The results clarified that the molecular characteristics of the removed tumor influence the final composition of the secreted seroma. Specifically, MIP-1a, MIP-1b, IP-10, IL-6, G-CSF, monocyte chemoattractant protein1- monocyte chemotactic and activating factor (MCP1-MCAF), and osteopontin were expressed higher in more aggressive tumors. Furthermore, differential expression of several small molecules was detected in the seroma of BC patients with mastectomy or quadrantectomy. In mastectomized patients, IL-1ra, IL-1b, IFN-γ, IL-6, G-CSF, osteopontin, IP-10, and MIP-1b were significantly higher than in quadrantectomized patients (109). The quantitative molecular diagnosis of cytokeratin-19 (CK19) and carcinoembryonic antigen (CEA) that target cancer cells in axillary seroma showed that they are a predictor of locoregional recurrence in mastectomized BC patients (110). In pioneering research, Belletti et al. compared non-IORT-seroma with IORT-seroma and revealed that TARGIT might possess an anti-tumor effect and surpass cancer cell kill *via* radiation therapy through altering the cytokines and growth factors existing in the resection cavity. They evaluated the proteomic content of seromas and detected that in seroma derived from TARGIT-treated patients compared to non-treated ones, 10 proteins enhanced while 20 proteins decreased (12). Kulcenty et al. conducted a quantitative investigation of the composition of seroma in patients with BC subtypes of luminal A and luminal B and between two groups of non-treated and treated with IORT. The comparison showed that TNF-beta, macrophage migration inhibitory factor (MIF), IL-7, IL-8, and IL-13 were significantly reduced in IORT-seroma; However, these findings were obtained without a differential diagnosis of molecular subtypes in the seroma groups. Moreover, enhanced concentrations of G-CSF, cutaneous T-cell-attracting chemokine (CTACK), IL-1 beta, hepatocyte growth factor (HGF), and TNF-alpha were characterized in IORT-seroma. They found that several cytokines were overexpressed in the luminal A subtype in the IORT-treated group, which may have anti-tumor characteristics (111). In a recent study, Wuhrer et al. analyzed seromas collected 24h after breast-conserving surgery (from 42 patients) with and without IORT treatment and observed dramatic changes in populations of immune cells and levels of cytokine (112). None of the investigated subpopulations, such as Treg, T cells, and myeloid cells, showed alteration in their activation states or their counts in cellular fraction analysis of the seroma and blood samples of the patients 24 h after IR treatment compared to control. Moreover, both groups did not alter the leucocyte fraction’s apoptosis rate. Thus, IORT did not affect the processes in cellular immunity during the first 24h after surgery in the local environment. In this study, levels of cytokines in seroma were significantly changed in the IORT-treated group; results showed that cytokines including GRO-α,oncostatin-M, and IL-1β are reduced while Leptin is enhanced with IORT treatment. All of these cytokines are linked to inflammation and tumor growth. **Figure 4** summarizes the studies related to seroma composition regarding protein changes.

#### 1.3.2 Effects of seroma on breast cancer cell lines; IORT vs non-IORT

Tagliabue and collaborators first described the proliferative effects of seroma on cultures of BC cells, who tested 24h post-surgical seroma and serum from 13 BC patients on SKBR-3, MDA-MB-453, MDA-MB361, MDA-MB231, MDA-MB-435, MCF- 7 cell lines. The results clarified that all of the cell lines were stimulated to proliferate in response to the drainage fluids, although the HER2-positive cell lines showed more proliferation levels than the HER2-negative ones. Their findings showed that seroma and post-surgical serum samples comprised growth factors capable of inducing the proliferation of HER-2-positive breast cancers. Although surgical wounds provided favorable conditions for the proliferation of tumor cells, carcinomas with overexpression of HER-2 revealed a higher rate of stimulating growth. It suggests several factors secreted during repair and healing are particularly active in inducing the HER-2-positive cells (113). In their several studies, Belletti and colleagues highlighted that collected seroma from BC patients within 24 hours after surgery plays a principal role in the proliferation, survival, and motility of BC cells (12, 114, 115). Segatto et al. found that the post-surgical collected seroma highly stimulates mammosphere formation in BC cells. The researchers used EGF (as a standard stimulative agent) and seroma on cell lines of MDA-MB-231, BT-474, MDA-MB-468, and MCF-7 to test mammosphere formation. The seroma-stimulated cell lines showed a higher mammosphere forming efficacy (MFE) than those induced with EGF. Seroma highly activates signal transducer and activator of transcription 3 (STAT3) in BC cell lines. The STAT3 affects the proliferative phenotype of BC cells, and its signaling is essential for the self-renewal ability of the seroma-induced cells (116). Another study on the effects of seroma on BC cell lines by Ramolu et al. showed the capability of three types of seroma to induce the proliferation of BC cell lines. They collected seroma from 30 patients who had tumor surgery (10 patients) or underwent induction chemotherapy after tumor surgery (10 patients) or breast reconstruction (10 patients). The seromas were used to grow MCF-7 and HCC1937 cell lines. The results showed that all three groups of seromas induced the proliferation of the cells.

Interestingly, the proliferation index from culturing HCC1937 cells was significantly higher than MCF-7 cells, suggesting more sensitivity of triple-negative cell lines to stimulation by seroma (117). In studies by Belletti et al. and, Herskind et al., seroma obtained from patients treated with IORT led to more reduced invasion and proliferation of BC cell lines *in vitro* compared to those induced by seroma from non-IORT patients. However, in the short-term 2D cell culture of BC cell lines with molecular types of ER/PR, -Her2/neu, and ER/PR−, Her2/neu+, IORT had no significant effect on the proliferative capacity of seroma; although, it showed the significant effects on invasion assay on 3-D Matrigel and migration test (12, 13). To confirm the results of previous studies, Veldwijk and collaborators evaluated the clonogenic and long-term proliferation effects of IORT-seroma and non-IORT-seroma on the MCF-7 cell line. Their results showed that the difference between these groups was insignificant and that the cells required 3% FBS in addition to seroma for short-term and clonogenic proliferation (118). Recently, Agresti et al. treated MDA-MB-231, MCF-7, SKBR-3, HCC1937, BT-549, and T-47D with post-surgery seroma (collected 24h after surgery) from 27 BC patients. Measurement of cell growth in 2D culture over 4 days showed that seroma stimulated robust cell proliferation and migration in all cell lines (109).

Zaleska et al. treated 8 BC cell lines with seroma collected from conservative−breast surgery (WF) and compared data to that of seroma from IOERT treatment RT-WF (≤10 Gy) for 4 days to indicate the effect of seromas on the phenotype of cancer stem cells. Then, the differentiation cluster of CD44+/CD24-/low phenotype and activity of aldehyde dehydrogenase 1 (ALDH1) were characterized. Each of the two types of fluids impacted the CD44+/CD24-/low phenotype. They showed different consequences between cell lines, even in histologically similar subtypes. RT−WF led to the decreased CD44+/CD24-/low population in basal−like MDA−MB−468 and BT−549, while the two fluids inhibited these populations in the luminal type MCF7 cell line. The HER2 −overexpressing subtypes protected a minimal population of CD44+/CD24-/low, but the two postoperative fluids stimulated the growth of SK−BR−3. Compared to RT−WF, WF showed a more substantial effect on ALDH1 activity. Depending on the histological subtype of the cell lines, a different stimulatory effect was observed. The most robust stimulation was in the control group for the luminal subtypes with low dehydrogenase activity (119).

In a recent study, Kulcenty and colleagues published reports about the effects of IORT-seroma on BC cells. To evaluate the marker expression related to extrinsic and intrinsic apoptosis pathways, they incubated MCF-7 cell lines with IORT-seroma and non-IORT-seroma from BC patients for 4 days. Their result indicated the activation of the extrinsic apoptosis pathway by IORT-seroma (120). To clarify bystander effects of IORT-seroma on BC cells, they incubated MDA-MB-468 and MCF-7 cell lines with non-IORT-seroma, IORT-seroma from 16 patients, and conditioned media (CM) from irradiated cells. They measured the level of apoptosis induction, the rate of breaks in double-strand DNA, and the alterations in DNA repair-associated gene expression. They found that despite the induction by non-IORT-seroma, the induction by IORT-seroma and non-IORT-seroma+CM stimulated the double-strand breaks and enhanced the expression of DNA repair-associated genes (121). They incubated MDA-MB-468 and MCF-7 cell lines with non-IORT-seroma and IORT-seroma to determine the underlying mechanisms leading to the reduced tumorigenic potential of IORT-seroma and confirm its effect on the activation of bystander effects in cell lines. The phenotype modification of CSCs in the EMT process was investigated to determine the inductive migration effect of seroma on BC cells. Their results showed that seroma triggers the phenotype of CSC and EMT process in BC cell lines; however, its impact was partly questioned when incubated with IORT-seroma. In addition, the radiation-stimulated bystander effect’s role in changing WF properties to persuade the EMT process and CSC phenotype formation was confirmed (122). To compare the biological effects of seroma and IORT-seroma on non-irradiated neighbors of the cancer cells (bystander effects), the MDA-MB-468 cell line was treated with non-IORT-seroma, IORT-seroma, and CM derived from irradiated cells. Then, the microarray analysis was carried out. The analysis showed that IORT-seroma and non-IORT-seroma+RIBE groups have a similar effect on the same biological processes, such as enhancing cell-cycle regulation, oxidative phosphorylation, and DNA repair. The non-IORT-seroma group has its effect through over-activation of the involved pathways on the inflammatory response, INF-α and INF-γ response, and the signaling pathway of IL6 JAK/STAT3. These results showed that MDA-MB-468 cells induced by IORT-seroma and cells stimulated with non-IORT-seroma plus RIBE share common biological processes (123). A recent study on IORT-seroma and non-IORT-seroma on MDA-MB-231 and mesenchymal stromal cells clarified that seroma from IORT-treated patients affected the MSC behavior and modified the secretome of these cells. After 34h, IORT-seroma inhibits the proliferation of the MSCs with a similar method and kinetics related to the MSC’s doubling time (30–40 h). Overall, these studies provide the results that IORT alters the factor composition of seroma, which decreases the proliferation of the MSCs, the capacity of wound healing, and the activity of chemotactic migration.

**Figure 5:**
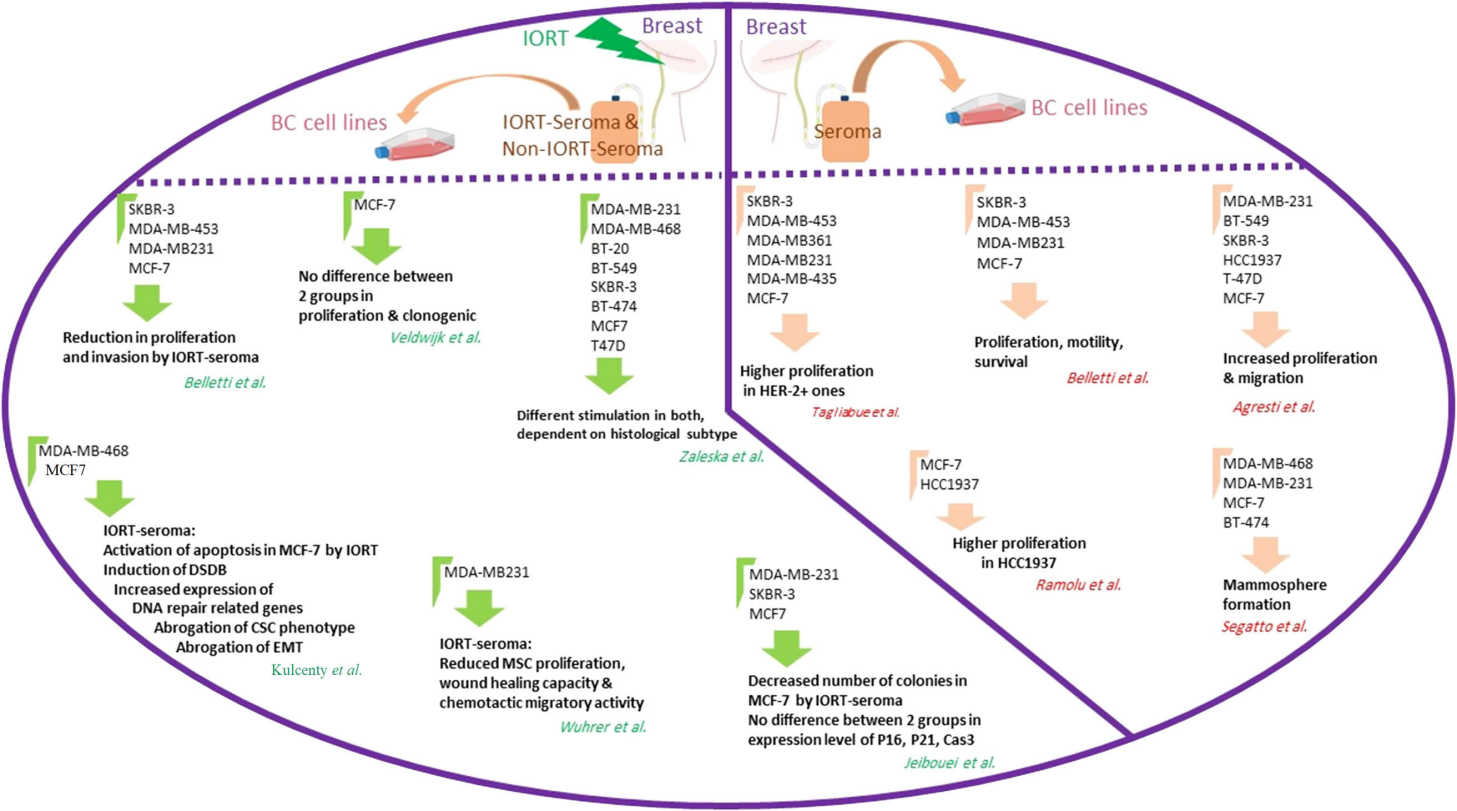
The studies related to the effects of seroma on BC cell lines. The right part of the circle shows the results from the effects of breast cancer seroma on breast cancer cell lines. The left part of the circle shows the results of the effects of IORT-treated and non-IORT-treated breast cancer seroma on breast cancer cell lines.

Moreover, analysis of MSCs-CM cultured in 0.5% IORT- and control-seroma and collected after 72h showed significantly decreased RANTES, GRO-α, and VEGF in the IORT-seroma group (112). To confirm the tumor inhibitory effects of IORT-seroma compared with non-IORT-seroma, we evaluated migration, proliferation, viability, and invasion in three BC cell lines. The viability and proliferation results clarified that MDA-MB-231 cells benefit more than SKBR-3 and MCF-7 from the anticancer effects of IORT-seroma. The findings of the clonal survival assay in MCF-7 cells showed that the number of colonies was reduced in IORT-seroma-treated cells compared with the other groups. IORT-seroma-treated and non-IORT-seroma-treated cells showed no significant change in expression levels of proteins associated with cell cycle arrest (P16, P21) and the expression level of Caspase 3. Furthermore, our results confirmed the previous findings about tumor progressive effects of seroma on these three BC cell lines (124). **Figure 5** presents the studies related to the effects of seroma on BC cell lines.

#### 1.3.3 Effects of seroma on breast cancer primary cells; IORT vs non-IORT

Most data indicate post-surgery seroma strongly induces proliferative and aggressive phenotypes in BC cell lines. To achieve more reliable results, these findings *in vivo* outcomes are required. Zhang et al. cultured primary cells from BC cells with or without seroma and then treated the cells with different anticancer drugs. Generally, a remarkable enhancement in survival rates was observed in the seroma-treated cells compared to the non-treated cells among different subgroups of the various anticancer drugs. The BC cells treated with seroma collected from premenopausal patients displayed a significantly higher rate of survival compared to those of the control group in all anticancer drugs. Finally, seroma-treated primary BC cells reported higher resistance to chemotherapy drugs (125). To mimic the tumor’s *in vivo* microenvironment and re-evaluate previous *in vitro* effects of seroma on breast tumor cells, we designed a 3D model using human-derived specimens. Spheroids from 23 breast tumors were cultured in the collagen matrix in microfluidic devices. Spheroids derived from each patient were treated for six days with the 24h seroma collected from the patients. Final data from fluorescent live/dead staining on day 6 showed that in 22 samples, the percentage of live cells was significantly higher in seroma-treated samples compared to cells treated with Roswell Park Memorial Institute (RPMI) (as a control for each sample) (124).

**Figure 6:**
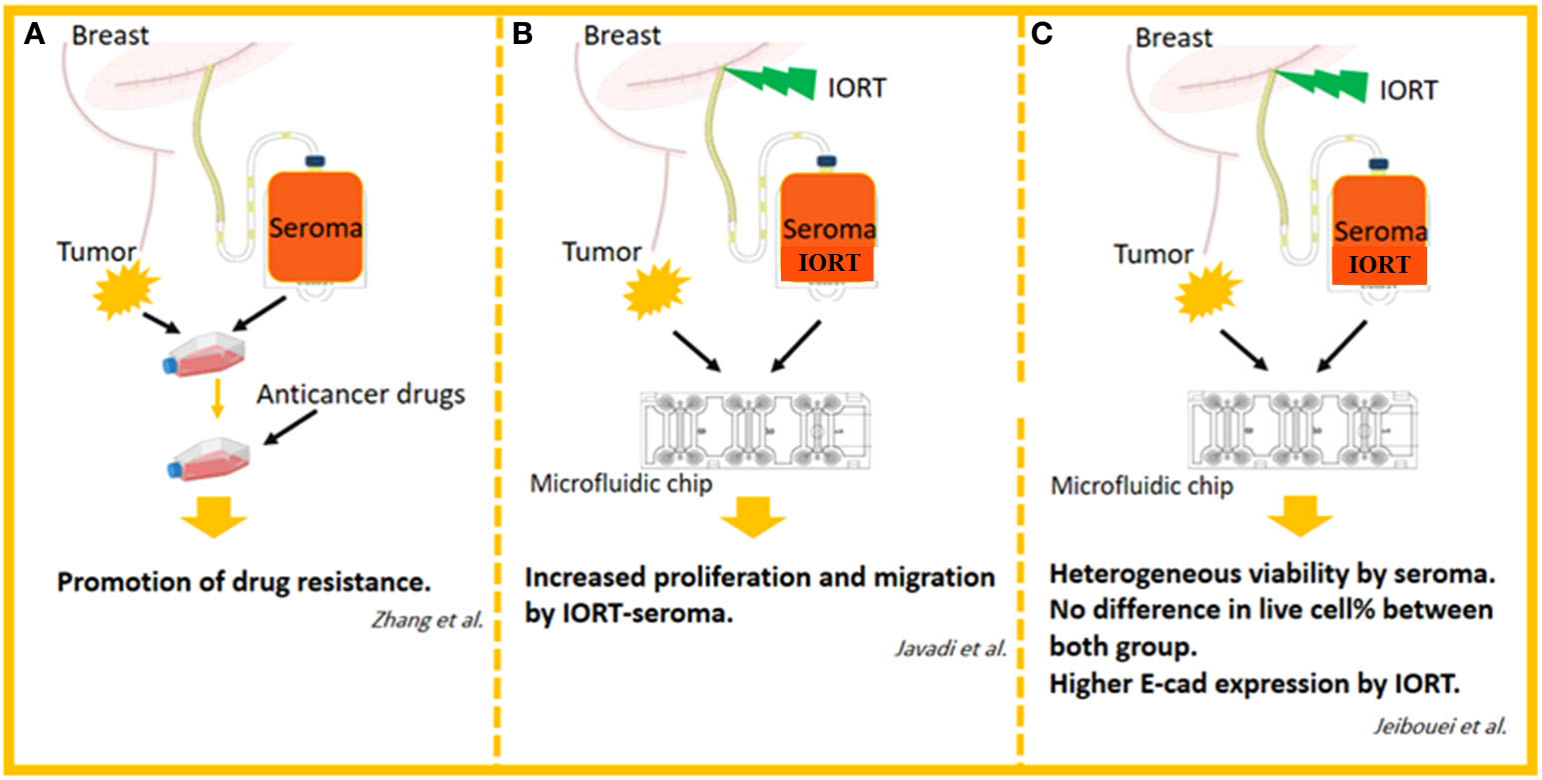
The studies related to the effects of seroma on primary BC cells. **(A)** Effects of drugs on seroma-treated human-derived breast cancer cells in 2D cell culture system. **(B)** Effects of IORT-treated and non-IORT-treated seroma on human-derived breast tumor spheroids in 3D microfluidic chips (visual evaluation of proliferation and migration). **(C)** Effects of IORT-treated and non-IORT-treated seroma on human-derived breast tumor spheroids in 3D microfluidic chips (visual and molecular evaluation of proliferation, apoptosis, migration, and heterogeneity).

Interestingly, one sample displayed the opposite result. We concluded that, however, most BC patients take advantage of removing seroma, the effects of seroma on tumor progression may not show a similar effect in all patients, and it can depend on many unknown factors (126). In another study, we assessed the radiobiological impact of IORT-seroma on human-derived specimens in a 3D model mentioned above. No significant difference in the percentage of live cells was observed between IORT-seroma-treated specimens with non-IORT-seroma-treated specimens after six days of treatment. The caspase 3 and E-cadherin expression levels in these specimens showed that despite similar caspase 3 in both groups, IORT-seroma-treated spheroids showed a higher level of E-cadherin compared to non-IORT-seroma-treated spheroids. It is worth noting that in both IORT-seroma and non-IORT-seroma groups, the expression levels of both E-cadherin and Caspase 3 were significantly higher in seroma-treated spheroids compared to RPMI-treated spheroids (as control). This study suggested IORT-seroma as a fluid containing inhibitory factors for tumor migration in the microfluidic system (124). Also, we showed increased proliferative and migrative characteristics of spheroids from four BC patients under IORT-seroma treatment using time-lapse imaging (127). **Figure 6** presents studies on seroma’s effects on primary BC cells.

## 2 Conclusion

Suction drainage placement after BCS is popular to prevent seroma formation in BC cases. However, it has some distinct drawbacks, such as an infection caused by the retrograde entry of skin bacteria through the drain, patient discomfort due to drain placement, and a need for daily nursing at home. Moreover, policies of drain removal are broadly different across various BC centers. Several studies have explored the safety of early drain removal according to multiple clinical endpoints. Studies revealed that seroma acts as a stimulative factor in tumor development through its interaction with cytokines, chemokines, and MMPs. According to data indicating beneficial direct and indirect effects of IORT on BC patients, some researchers assumed that IORT-induced seroma might mediate a part of these therapeutic effects of IORT. However, many studies such as analysis of IORT-seroma composition, treatment of BC cell lines and human tumor tissues, and assessment of their behavior under treatment of the collected seroma in 2D and 3D systems revealed that IORT-seroma has the same results as non-IORT-seroma in tumor cavity after the surgery.

The tumor heterogeneity could be a role player in the effectiveness of seroma on tumor behavior. Furthermore, in a 3D microfluidic study, we observed that heterogeneity of tumor and seroma have different effects in different patients. Our proteomic and transcriptomic data from tumor bed analysis also showed that IORT could affect tumor bed and probably remain cancer cells in tumor margins through immune system infiltration. Overall, evidence indicates that studies on seroma or IORT could not discover their mechanisms of tumor inhibition because of the variation in body reactions of patients. It seems that it is related to the immune system and probably unknown or unstudied factors in this area, such as microbiota in the body of patients. Deciphering mechanisms associated with immune system infiltration and abscopal effects consider personalized medicine by using profiling to address the questions about the inhibiting effects of IORT. In conclusion, we cannot provide a rationale for preserving or removing seroma in IORT-treated BC patients. The question of whether IORT-seroma has a beneficial effect can only be answered in a trial with a clinical endpoint, which needs to be investigated.

## Author contributions

The concept of the manuscript was conceived by MA and HZ. SJ drafted the manuscript, and the other authors made direct intellectual and substantial contributions to the work, and accepted the manuscript for publication. All authors contributed to the article and approved the submitted version.

## Acknowledgments

Authors thank Dr. Ebrahim Mostafavi for his unconditional support. Authors apologize to the scientists whose helps to IORT and personalized medicine in breast cancer could not be acknowledged because of space limitations.

## Conflict of interest

The authors declare that the research was conducted in the absence of any commercial or financial relationships that could be construed as a potential conflict of interest.

## Publisher’s note

All claims expressed in this article are solely those of the authors and do not necessarily represent those of their affiliated organizations, or those of the publisher, the editors and the reviewers. Any product that may be evaluated in this article, or claim that may be made by its manufacturer, is not guaranteed or endorsed by the publisher.
